# Does the reduction of inferior turbinate affect lower airway functions?^☆^

**DOI:** 10.1016/j.bjorl.2017.10.007

**Published:** 2017-11-06

**Authors:** Ozlem Unsal, Mehtap Ozkahraman, Mufide Arzu Ozkarafakili, Meltem Akpinar, Arzu Yasemin Korkut, Senem Kurt Dizdar, Berna Uslu Coskun

**Affiliations:** a Sisli Hamidiye Etfal Training and Research Hospital, Clinic of Otorhinolaryngology, Istanbul, Turkey; b Sisli Hamidiye Etfal Training and Research Hospital, Clinic of Pulmonary Diseases, Istanbul, Turkey

**Keywords:** Acoustic rhinometry, Turbinates, Hypertrophy, Spirometry, Respiratory system, Rinometria acústica, Conchas nasais, Hipertrofia, Espirometria, Sistema respiratório

## Abstract

**Introduction:**

Although the nose and lungs are separate organs, numerous studies have reported that the entire respiratory system can be considered as a single anatomical and functional unit. The upper and lower airways affect each other either directly or through reflex mechanisms.

**Objective:**

In this study, we aimed to evaluate the effects of the radiofrequency ablation of persistent inferior turbinate hypertrophy on nasal and pulmonary function.

**Methods:**

Twenty-seven patients with bilateral persistent inferior turbinate hypertrophy without septal deviation were included in this study. All of the patients were evaluated using anterior rhinoscopy, nasal endoscopy, acoustic rhinometry, a visual analogue scale, and flow-sensitive spirometry on the day before and 4 months after the radiofrequency ablation procedure.

**Results:**

The post-ablation measurements revealed that the inferior turbinate ablation caused an increase in the mean cross-sectional area and volume of the nose, as well as in the forced expiratory volume in 1 s, forced vital capacity, and peak expiratory flow of the patients. These differences between the pre- and post-ablation results were statistically significant. The post-ablation visual analogue scale scores were lower when compared with the pre-ablation scores, and this difference was also statistically significant.

**Conclusion:**

This study demonstrated that the widening of the nasal passage after the reduction of the inferior turbinate size had a favorable effect on the pulmonary function tests.

## Resumo

**Introdução:**

Embora o nariz e os pulmões sejam órgãos separados, numerosos estudos relataram que todo o sistema respiratório pode ser considerado como uma única unidade anatômica e funcional. As vias aéreas superiores e inferiores afetam uma à outra diretamente ou através de mecanismos reflexos.

**Objetivo:**

Avaliar os efeitos da ablação por radiofrequência em conchas nasais inferiores com hipertrofia persistente sobre a função nasal e pulmonar.

**Método:**

Foram incluídos neste estudo 27 pacientes com hipertrofia persistente bilateral de conchas inferiores sem desvio septal. Todos os pacientes foram avaliados com rinoscopia anterior, endoscopia nasal, rinometria acústica, escala visual analógica e espirometria sensível ao fluxo no dia anterior e quatro meses após o procedimento de ablação por radiofrequência.

**Resultados:**

As medidas pós-ablação demonstraram que a ablação das conchas nasais inferiores resultou em um aumento da área transversal média e do volume do nariz, bem como do volume expiratório forçado em um segundo, da capacidade vital forçada e do fluxo expiratório máximo dos pacientes. Essas diferenças entre os resultados pré e pós-ablação foram estatisticamente significantes. Os escores da escala visual analógica pós-ablação foram menores quando comparados com os escores pré-ablação e essa diferença também foi estatisticamente significante.

**Conclusão:**

O alargamento da passagem nasal após a redução do tamanho das conchas nasais inferiores teve efeito favorável nos testes de função pulmonar.

## Introduction

Inferior Turbinate Hypertrophy (ITH) is a common cause of chronic nasal obstruction.^1^, ^2^ Although this enlargement is mostly reversible, it can persist, as in cases of vasomotor rhinitis, allergic rhinitis, or compensatory hypertrophy, because of septal deviation.^3^ Many different conservative ITH therapies have been applied, including the use of antihistamines, systemic decongestants, or intranasal sprays.^4^, ^5^ However, surgical reduction may be a good option in those cases in which medical treatment has failed, and it is the most effective treatment for ITH.^6^ Radiofrequency Ablation (RFA) is widely used for the surgical reduction of inferior turbinate's, although there is a lack of consensus regarding the optimal surgical technique.^7^, ^8^, ^9^

Even though the nose and lungs are usually treated as separate entities, the upper and lower airways can be regarded as a single anatomical and functional unit, since they directly affect each other.^4^ The treatment of sinonasal pathologies, including allergic rhinitis, sinusitis, nasal polyps, and septal deviation, helps to resolve not only nasal obstruction, but can also positively influence lower airway functions.^10^, ^11^, ^12^, ^13^ For this research, we aimed to evaluate the effects of the treatment of persistent ITH on nasal and pulmonary functions.

## Methods

This prospective study was performed between June 2015 and December 2016, and was approved by the Institutional Ethical Committee (approval protocol number 238). Informed consent was obtained from all of the volunteers. Twenty-seven adult patients (21 males, 6 females), between the ages of 20 and 59 years old (32.16 ± 10.48), who had been suffering from nasal obstruction due to bilateral ITH for at least 1 year, and who had been using nasal steroids for at least 3 months, were enrolled in this study.

The medical histories of the participants were recorded, and the standardized American Thoracic Society respiratory disease questionnaire for adults was used to assess their respiratory symptoms.^14^ Those patients with chronic obstructive pulmonary disease, asthma, allergic rhinitis, malignancy, cardiac disease, and blood coagulation pathologies, as well as active and ex-smokers, and those patients using systemic steroids, antileukotriene, nedocromil, teofilin, and anticoagulants were excluded from this study. Nasal pathologies, such as septal deviation, nasal valve disorders, alar collapse, septal perforation, middle turbinate hypertrophy, and nasal polyposis, and a previous history of nasal surgery, were also accepted as exclusion criteria.

An endoscopic examination was performed in each patient with a 4 mm, 0° endoscope (Karl StorzGmbh Co., *Tuttlingen*, Germany). A 10 point Visual Analogue Scale (VAS) (1 being the least, 10 being the most) was used to determine the severity of the nasal obstruction. This scale was used before and 4 months after the RFA of the inferior turbinates.

All of the patients underwent Acoustic Rhinometry (AR) using a Rhinoscan SRE2000 (RhinoMetrics A/S, Lynge, Denmark) in the sitting position before and 4 months after the RFA. No decongestants were used before the test, and each nasal passage was tested separately. The measurements were taken by the same researcher. A round plastic nosepiece with a 13 mm inner diameter was used for the test. The patient was asked to hold his or her breath after the nasal adaptor was placed. Mean cross-sectional area (MCA) 1 and 2 were the distance from the nostrils to 2.2 cm and from 2.2 to 5.4 cm, respectively. Volumes 1 and 2 were measured from 0 to 2.2 cm and 2.2 to 5.4 cm, respectively, while the total volume was measured using the total distance (from 0 to 5.4 cm). For each patient, we repeated the AR measurements at least thrice (both pre- and post-operatively) and recorded the average values.

**Figure 1:**
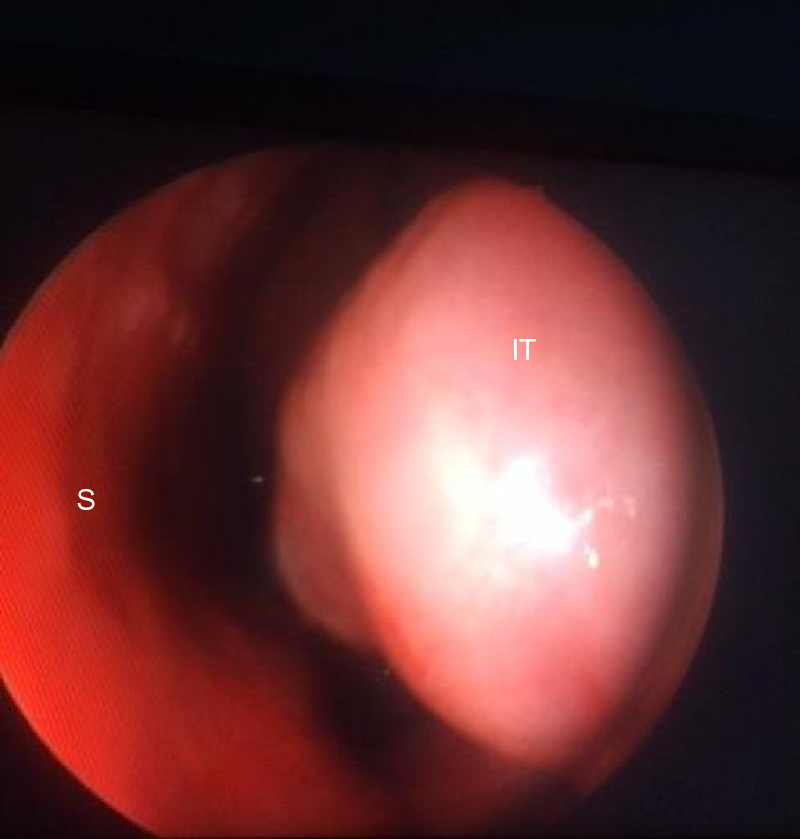
An endoscopic image of the left nasal cavity. Hypertrophic inferior turbinate of a patient before radiofrequency ablation is demonstrated. S, Septum; IT, Inferior Turbinate.

**Figure 2:**
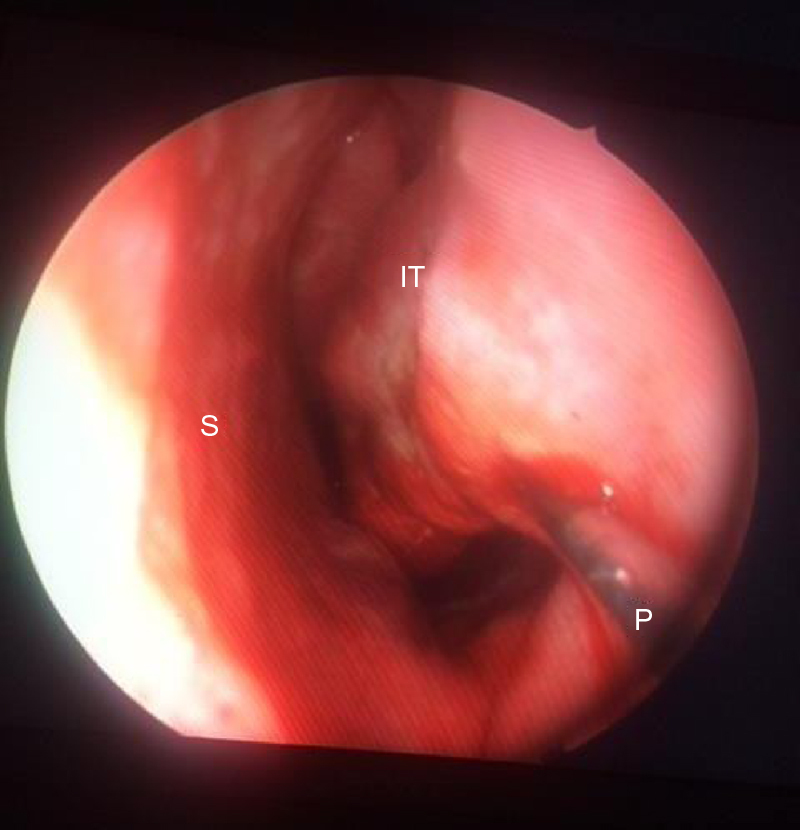
An image showing the reduction of left inferior turbinate of a patient using radiofrequency ablation technique. A conchal probe is inserted into the submucosal plane of inferior turbinate, and posterior, middle and anterior one thirds of the turbinate are ablated by a total of 300 J of energy. S, Septum; IT, Inferior Turbinate; P, Conchal probe.

All of the surgeries were performed by a senior surgeon under local anesthesia using the same technique. In each patient, topical lidocaine spray was applied to both mucosal surfaces. After 5 min, 2 mL of 40 mg lidocaine HCl with 0.025 mg adrenalin diluted with 2 mL of 0.9% NaCl was injected into each inferior turbinate using a 22 G × 32 mm dental injector. The regulation and monitoring of the entire soft-coagulation process were conducted via a radiofrequency generator (CelonLabENT; Celon AG, Teltow, Germany). A conchal probe (CelonProBreath, Celon AG, Teltow, Germany) was inserted into the submucosal plane of the anterior, middle, and posterior one-third of each inferior turbinate under endoscopic guidance (Figure 1, Figure 2). The energy supplied for one shot was 12 W, and a total of 300 J of energy was delivered to each turbinate for 8–9 s. No additional out-fracturing was performed for the turbinoplasty.

Each of the patients was invited for a follow-up examination 1 week and 4 months after the procedure. Specific attention was given to the existence of any crust, infection, dryness, and/or odor. The crusts or secretions in both nasal cavities were removed before the AR measurement control and Pulmonary Function Test (PFT). The PFT was performed using a Spirolab III spirometer (MIR, Rome, Italy) based on the American Thoracic Society recommendations.^15^ The Peak Expiratory Flow (PEF), Forced Expiratory Volume in 1 second (FEV1), Forced Vital Capacity (FVC), and ratio of the FEV1 to FVC (FEV1/FVC) were measured. The best value of three trials was recorded.

The statistical analysis was performed using SPSS version 15.0 for Windows. The descriptive statistics for the numerical variables were expressed as the mean and standard deviation. A paired *t*-test was used for the comparison of the numerical variables between the dependent groups when a normal distribution was shown; if not, the Wilcoxon test was used. Statistical significance was defined as *p* < 0.05.

## Results

Twenty-seven patients were included in the present study; with a mean age of 32.16 ± 10.48 years old (range 20–59). There were 6 females (22.2%) and 21 males (77.8%). RFA was applied to both inferior turbinates of each patient. In total, 54 nasal cavities were analyzed.

### Subjective evaluation of symptoms

The pre-ablation mean VAS score (8.58 ± 1.07) was compared with the post-ablation mean VAS score (3.11 ± 1.24) for the left nasal cavity and the pre-ablation mean VAS score (8.63 ± 1.07) was compared with the post-ablation mean VAS score (3.26 ± 1.37) for right nasal cavity. The descriptive statistics of the VAS results revealed that the pre-operative values were significantly higher than the post-surgical 4 month measurements for the left and right nasal cavities (*p* < 0.001 and *p* < 0.001, respectively).

### Objective evaluation of nasal cavities by acoustic rhinometry

**Figure 3:**
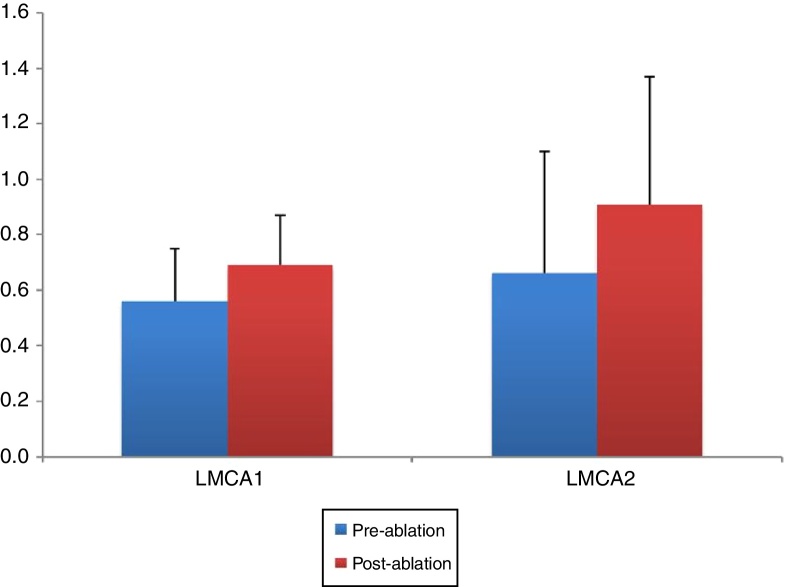
Demonstration of the changes in the left mean cross-sectional area 1 and 2 (LMCA 1, 2) before and after the reduction of inferior turbinate size. Data are presented as median, and minimum and maximum values. The increase in the LMCA 1 and 2 after the treatment of ITH was statistically significant (*p* < 0.001 and *p* = 0.001, respectively). LMCA 1 and 2, Left Mean Cross-sectional Area 1 and 2.

**Figure 4:**
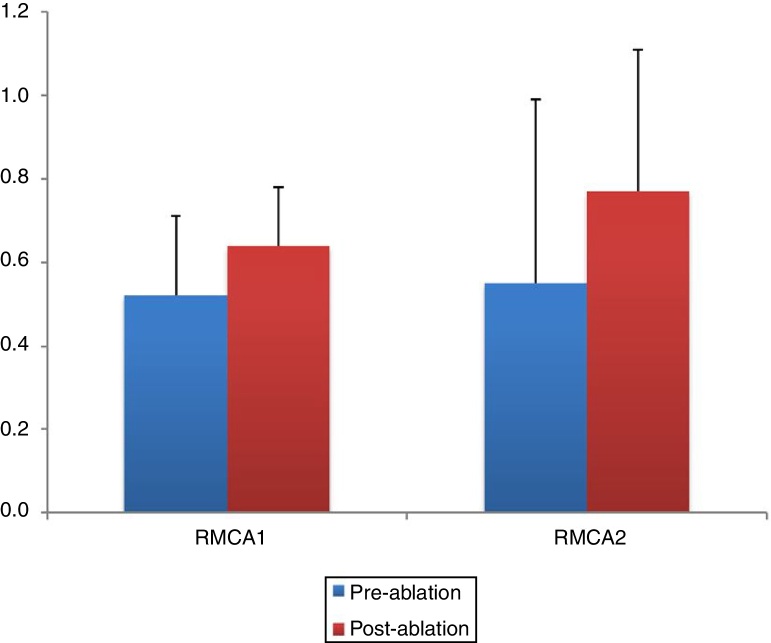
The post-operative values of RMCA 1 and 2 were compared with the pre-operative measurements. Statistically significant increase in the RMCAs was determined after reducing the inferior turbinate size (*p* < 0.001 and *p* < 0.001, respectively). RMCA 1 and 2, Right Mean Cross-sectional Area 1 and 2.

**Figure 5:**
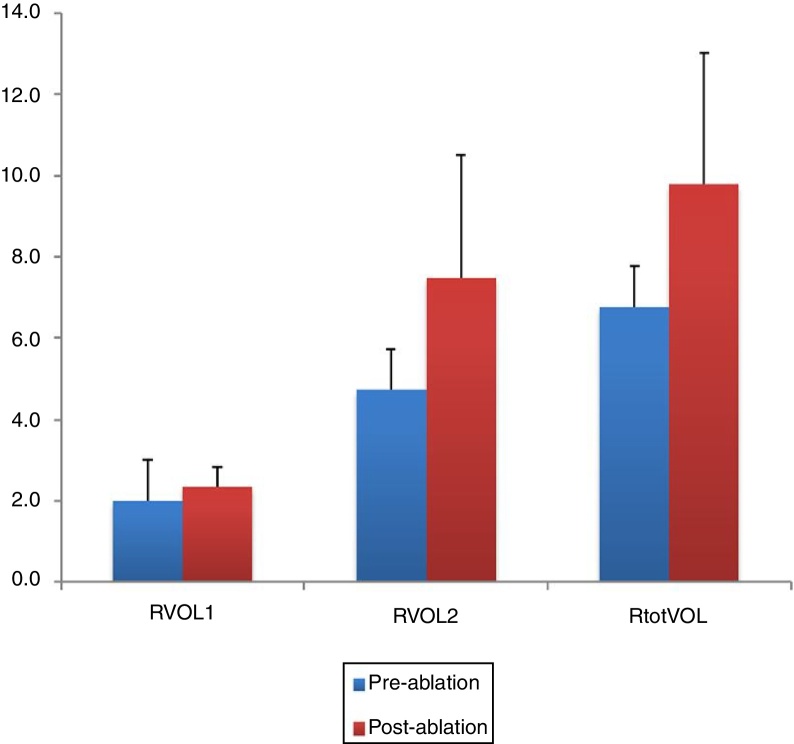
Illustration of the changes in right nasal volumes (RVOL 1, 2 and RtotVOL) before and after reduction of inferior turbinate. The treatment of ITH had positive effect with statistical significance on the all right nasal volumes (*p* = 0.003, *p* < 0.001 and *p* < 0.001, respectively). RVOL 1 and 2, Right nasal Volume 1 and 2; RtotVOL, Total Volume of the right nasal cavity.

**Figure 6:**
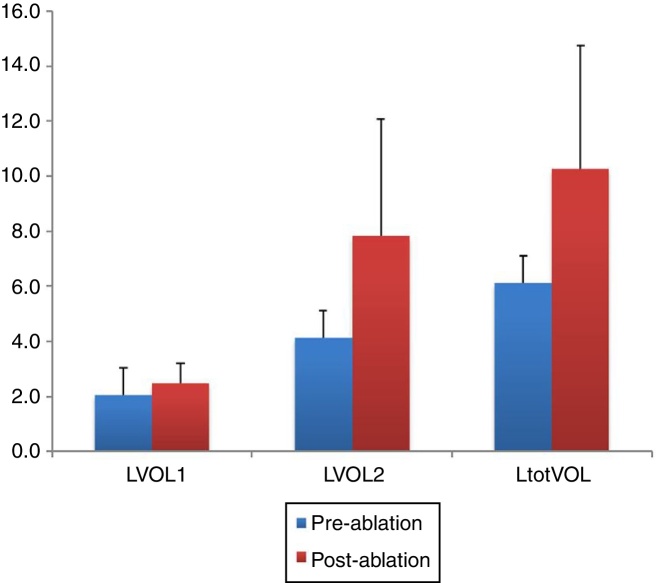
The changes in left nasal volumes (LVOL 1, 2 and LtotVOL) before and after reducing the inferior turbinate size were demonstrated. Statistically significant increase in all volumes of the left nasal cavity was determined after the treatment of ITH (*p* = 0.001, *p* < 0.001 and *p* < 0.001, respectively). LVOL 1 and 2, Left nasal Volume 1 and 2; LtotVOL, Total Volume of the left nasal cavity.

**Figure 7:**
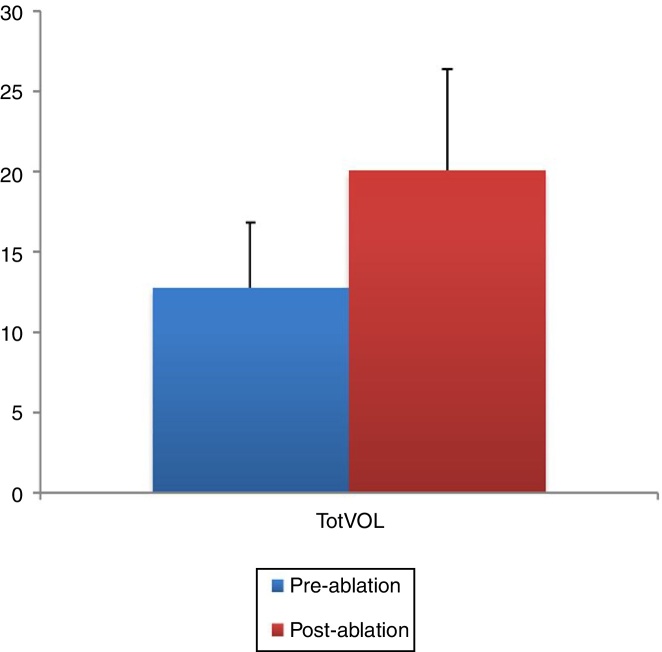
Demonstration of the Total Volume of the nose (totVOL) increased significantly after the treatment of ITH (*p* < 0.001). The entire nasal cavity expanded due to the reduction of the inferior turbinates. TotVOL, Total Volume of the nose (left total volume + right total volume).

The pre- and post-ablation MCAs and nasal volumes for the right and left nasal cavities were compared. As illustrated in Figure 3, Figure 4, the post-ablation left MCA 1 and 2 (LMCA 1, 2) and right MCA 1 and 2 (RMCA 1, 2) were higher when compared with the pre-ablation values, and the differences between them were statistically significant (*p* < 0.001, *p* = 0.001, *p* < 0.001, and *p* < 0.001, respectively). Similarly, the post-ablation right nasal volume 1 and 2 (RVOL 1, 2), Right total Volume (RtotVOL) (Fig. 5), left nasal volume 1 and 2 (LVOL 1, 2), Left total Volume (LtotVOL) (Fig. 6) and total volume of the nose (totVOL) (Fig. 7) were increased when compared with the pre-ablation volumes, and those differences were statistically significant (*p* = 0.003, *p* < 0.001, *p* < 0.001, *p* = 0.001, *p* < 0.001, *p* < 0.001, and *p* < 0.001, respectively).

### Evaluation of pulmonary function by flow-sensitive spirometer

**Figure 8:**
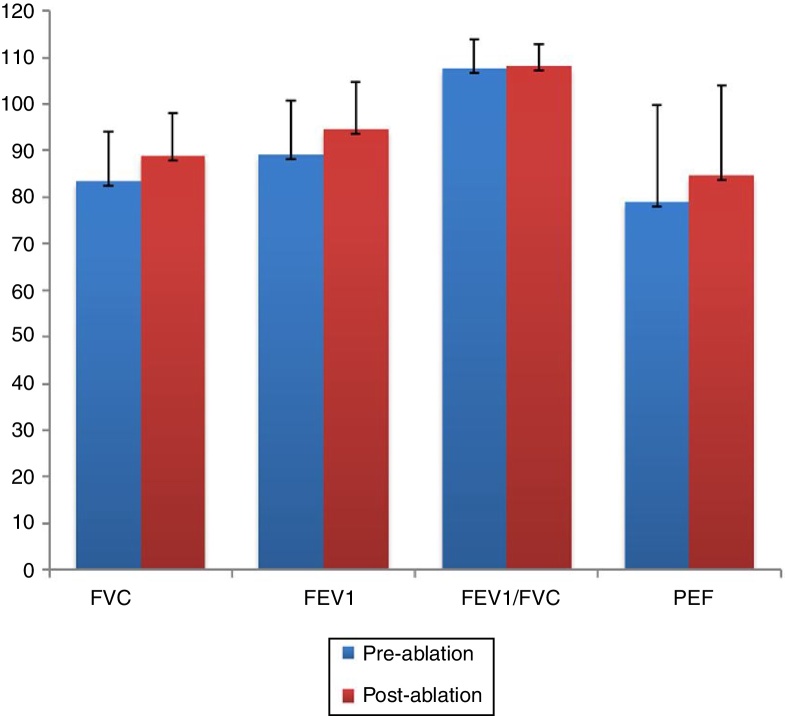
The spirometric measurements before and after the reduction of inferior turbinate. FVC, FEV1 and PEF evaluated 4 months after the treatment of ITH are determined to be increased with statistical significance (*p* < 0.001, *p* < 0.001 and *p* = 0.042, respectively). FVC, Forced Vital Capacity; FEV1, Forced Expiratory Volume in 1 second; PEF, Peak Expiratory Flow.

The post-ablation FVC, FEV1, and PEF results were found to be higher than the pre-ablation measurements, and the differences were statistically significant (*p* < 0.001, *p* < 0.001, and *p* = 0.042, respectively). However, when the pre- and post-ablation measurements of the FEV1/FVC ratio were compared, no marked difference was observed (*p* > 0.05) (Fig. 8).

## Discussion

For this research, the upper and lower airways were examined before and after the RFA of persistent ITH. The treatment of sinonasal pathologies, such as rhinosinusitis, allergic rhinitis, or septal deviation, has been previously investigated with regard to the effects on the pulmonary system. However, there were no studies regarding inferior turbinate and lung interactions found in the literature. Therefore, we aimed to evaluate the effects of inferior turbinate size reduction on both nasal and pulmonary functions. For this purpose, RF was preferred which is a widely used technique for reducing the size of the inferior nasal turbinates. This reduction results in an enlargement of the nasal airway and improves the flow volume of inhaled air.^16^

AR provides an objective measurement of the cross-sectional areas of the corresponding sections in the nose, and AR improvement after an RFA of the inferior turbinates has been reported in several studies.^7^, ^9^, ^17^, ^18^ In accordance with these reports, our study revealed that the inferior turbinate size was successfully reduced since all of the AR parameters were found to be significantly higher than the pre-ablation measurements. A statistically significant decrease was also observed in the post-ablation VAS scores when compared with the pre-ablation VAS scores of the patients. Overall, the reduction in the turbinate size enabled a widening of the nasal passages. In accordance with the AR results, the spirometric measurements (including the FEV1, FVC, and PEF) were improved after the reduction of inferior turbinate size, with statistical significance. However, it should be emphasized that these improvements in VAS scores, AR measurements and spirometric values indicate only the short term outcomes of the inferior turbinate reduction and the widening of the nasal passages due to the 4 month follow-up time. Additionally, these results do not allow to evaluate the effectiveness of the radiofrequency ablation technique that was used for reducing the size of the turbinate's in this study. A single technique was preferred for the treatment of ITH in our work. Therefore, other methods for the same purpose such as out-fracture, submucosal resection or bipolar cauterization of the inferior turbinate's may provide similar effects.

Due to the close relationship between the upper and lower airways, they can be regarded as a single anatomical and functional unit.^19^ The upper respiratory mucosa helps to humidify, heat, and filter the inhaled air, and thus protect the lower respiratory tract. Mucociliary clearance is important for protecting the mucosa against foreign particles, preventing bacterial infection, and moisturizing the epithelial cell layer. The humidity, mucociliary clearance, and temperature, which are the physiological conditions of the nasal cavity, are normally affected by the airstream, and can deteriorate in a decreasing airstream. The continuous airflow stimulation provides optimal nasal cavity aerodynamics, and facilitates the physiological function of the mucociliary activity.^20^ The conditioning and filtering capacity of the nose protects the lower airways and in the case of partial or complete loss of function of the nose, the dry or cold air and potentially harmful agents entering the bronchi can lead to bronchoconstriction.^21^ The rhinobronchial reflex which is thought to be one of the pathogenic mechanisms connecting the nose and lungs can be stimulated by inhalation of cold or unfiltered air and cause lower airway irritability and morbidities.^12^, ^22^

Also, the treatment of sinonasal pathologies may affect the lower airway, and vice versa. For instance, Karuthedath et al.^23^ evaluated the effects of endoscopic sinus surgery on the pulmonary function of patients with chronic rhinosinusitis, and they observed a remarkable recovery in the PFT results after the surgery. Likewise, a significant improvement in the PFT parameters after septoplasty was reported in the literature, and was attributed to normalizing the nasal airstream.^12^ In the present study, results compatible with these reports were found in the PFT parameters after the reduction of the inferior turbinates. The post-operative FEV1, FVC, and PEF were determined to be increased when compared with the pre-operative measurements. This may have been due to the improved nasal aerodynamics after the reduction of the inferior turbinate size.

The major limitation of this study was the inability to obtain a large number of cases due to the strict exclusion criteria. The selection of patients with an isolated ITH was quite difficult, since septal deviation is a frequently encountered nasal pathology. On the other hand, persistent ITH is most often seen in patients with allergic rhinitis. Other conditions, such as pulmonary and cardiac diseases, smoking, or the use of bronchoactive drugs, can affect the PFT parameters; therefore, these cases were also excluded from the study in order to observe only the effects of the inferior turbinate reduction on the pulmonary function. Additionally, the patients who left the study during follow-up also limited the number of participants.

## Conclusion

The upper and lower airways have not only anatomical continuity; there is also a strong interaction between them. Impaired nasal conditions and aerodynamics may lead to a loss of the protective function of the nose, and may activate sinonasal reflex mechanisms affecting the lower airway function. It has been reported that the treatment of nasal pathologies, such as septal deviation, chronic rhinosinusitis, or allergic rhinitis, creates an improvement in the lower airway function. However, the interactions between the surgical treatment of ITH and pulmonary functions have not been previously examined. This study revealed that the reduction in the inferior turbinate size provided significant improvement in the PFT results. Nevertheless, further studies using larger cohorts are needed to analyze these interactions.

## Funding

This research did not receive any specific grant from funding agencies in the public, commercial, or not-for-profit sectors.

## Conflicts of interest

The authors declare no conflicts of interest.

## References

[1] Bäck L.J., Hytönen M.L., Malmberg H.O., Ylikoski J.S. (2002). Submucosal bipolar radiofrequency thermal ablation of inferior turbinates: a long-term follow-up with subjective and objective assessment. Laryngoscope.

[2] Sapci T., Sahin B., Karavus A., Akbulut U.G. (2003). Comparison of the effects of radiofrequency tissue ablation, CO_2_ laser ablation, and partial turbinectomy applications on nasal mucociliary functions. Laryngoscope.

[3] Farmer S.E., Eccles R. (2006). Chronic inferior turbinate enlargement and the implications for surgical intervention. Rhinology.

[4] Lanier B. (2007). Allergic rhinitis: selective comparisons of the pharmaceutical options for management. Allergy Asthma Proc.

[5] Nassef M., Shapiro G., Casale T.B., Respiratory and Allergic Disease Foundation (2006). Identifying and managing rhinitis and its subtypes: allergic and nonallergic components – a consensus report and materials from the Respiratory and Allergic Disease Foundation. Curr Med Res Opin.

[6] Hol M.K., Huizing E.H. (2000). Treatment of inferior turbinate pathology: a review and critical evaluation of the different techniques. Rhinology.

[7] Gindros G., Kantas I., Balatsouras D.G., Kaidoglou A., Kandiloros D. (2010). Comparison of ultrasound turbinate reduction, radiofrequency tissue ablation and submucosal cauterization in inferior turbinate hypertrophy. Eur Arch Otorhinolaryngol.

[8] Cavaliere M., Mottola G., Iemma M. (2005). Comparison of the effectiveness and safety of radiofrequency turbinoplasty and traditional surgical technique in treatment of inferior turbinate hypertrophy. Otolaryngol Head Neck Surg.

[9] Demir U., Durgut O., Saraydaroglu G., Onart S., Ocakoglu G. (2012). Efficacy of radiofrequency turbinate reduction: evaluation by computed tomography and acoustic rhinometry. J Otolaryngol Head Neck Surg.

[10] Ragab S., Scadding G.K., Lund V.J., Saleh H. (2006). Treatment of chronic rhinosinusitis and its effects on asthma. Eur Respir J.

[11] Dahl R., Nielsen L.P., Kips J., Foresi A., Cauwenberge P., Tudoric N. (2005). Intranasal and inhaled fluticasone propionate for pollen-induced rhinitis and asthma. Allergy.

[12] Bulcun E., Kazkayasi M., Ekici A., Tahran F.D., Ekici M. (2010). Effects of septoplasty on pulmonary function tests in patients with nasal septal deviation. J Otolaryngol Head Neck Surg.

[13] Kountakis S.E., Bradley D.T. (2003). Effect of asthma on sinus computed tomography grade and symptom scores in patients undergoing revision functional endoscopic sinus surgery. Am J Rhinol.

[14] Comstock G.W., Tockman M.S., Helsing K.J., Hennesy K.M. (1979). Standardized respiratory questionnaires: comparison of the oldwith the new. Am Rev Respir Dis.

[15] (1987). Standardization of spirometry – statement of the American Thoracic Society – 1987 update. Am Rev Respir Dis.

[16] Leong S.C., Farmer S.E., Eccles R. (2010). Coblation^®^ inferior turbinate reduction: a long-term follow-up with subjective and objective assessment. Rhinology.

[17] Passali D., Loglisci M., Politi L., Passali G.C., Kern E. (2016). Managing turbinate hypertrophy: coblation vs. radiofrequency treatment. Eur Arch Otorhinolaryngol.

[18] Shah A.N., Brewster D., Mitzen K., Mullin D. (2015). Radiofrequency coblation versus intramural bipolar cautery for the treatment of inferior turbinate hypertrophy. Ann Otol Rhinol Laryngol.

[19] Ciprandi G., Caimmi D., Miraglia Del Giudice M., La Rosa M., Salpietro C., Marseglia G.L. (2012). Recent developments in united airways disease. Allergy Asthma Immunol Res.

[20] Shin S.H., Heo W.W. (2005). Effect of unilateral naris closure on the nasal and maxillary sinus mucosa in rabbit. Auris Nasus Larynx.

[21] Licari A., Castagnoli R., Denicolò C.F., Rossini L., Marseglia A., Marseglia G.L. (2017). The nose and the lung: united airway disease?. Front Pediatr.

[22] Passali D., Benedetto de F., Benedetto de M., Chiaravalloti F., Damiani V., Passali F.M. (2011). Rhino-Bronchial Syndrome. The SIO-AIMAR (Italian Society of Otorhinolaryngology, Head Neck Surgery-Interdisciplinary Scientific Association for the Study of the Respiratory Diseases) survey. Acta Otorhinolaryngol Ital.

[23] Karuthedath S., Singh I., Chadha S. (2014). Impact of functional endoscopic sinus surgery on the pulmonary function of patients with chronic rhinosinusitis: a prospective study. Indian J Otolaryngol Head Neck Surg.

